# Factors associated with maternal anaemia among pregnant women in Dhaka city

**DOI:** 10.1186/s12905-015-0234-x

**Published:** 2015-09-22

**Authors:** Hasina Akhter Chowdhury, Kazi Rumana Ahmed, Fatema Jebunessa, Jesmin Akter, Sharmin Hossain, Md. Shahjahan

**Affiliations:** Department of Biostatistics, Bangladesh University of Health Sciences, Dhaka, Bangladesh; Department of Health Promotion and Health Education, Bangladesh University of Health Sciences, Dhaka, Bangladesh; Department of Biochemistry and Cell Biology, Bangladesh University of Health Sciences, Dhaka, Bangladesh; Department of Reproductive and Child Health, Bangladesh University of Health Sciences, Dhaka, Bangladesh; Department of Health Promotion and Health Education, Bangladesh University of Health Sciences, Dhaka, Bangladesh; Department of Public Health, Daffodil International University, Dhaka, Bangladesh

**Keywords:** Pregnant women, haemoglobin level, associated factors, maternal anaemia, Bangladesh

## Abstract

**Background:**

Maternal anaemia is a common problem in pregnancy, particularly in developing countries. The study was aimed at determining the factors associated with anaemia among a group of pregnant mothers who attended an antenatal clinic in Dhaka city.

**Methods:**

This cross-sectional study included 224 pregnant women, who visited the antenatal clinic of the Marie Stops, Dhaka. Demographic data and information on maternal age, gestational age, educational and income level, and socioeconomic status were collected from all the subjects. Haemoglobin status was measured to assess their anaemia. A qualified technician drew venous blood samples from them. The reference values of haemoglobin were categorized according to the World Health Organization (WHO) criteria as follows: normal (11 g/dL or higher), mild (10–10.9 g/dL), and moderate (7–9.9 g/dL). Mild and moderate levels of haemoglobin were defined as anaemic (haemoglobin levels of <11 g/dL). The SPSS software (Windows version 16.0. SPSS Inc, Chicago, USA) was used for analyzing data.

**Results:**

The mean (±SD) age of the subjects was 26.4 ± 2.81 years. Sixty-three percent of the subjects had normal level of haemoglobin, and 37 % were anaemic 26 % mild and 11 % moderate. Maternal anaemia was significantly associated with age (p = 0.036), education (p = 0.002), income (p = 0.001), living area (p = 0.031). Results of binary logistic regression analysis showed that maternal anaemia was also significantly associated with age (p = 0.006), educational status (primary to 8th grade, p = 0.004; secondary and above, p = 0.002), living area (0.022), and income (0.021).

**Discussion:**

A significant proportion of pregnant women were found anaemic. Most data showed education has animpact on awareness to use of health services and iron supplementation should be encouraged to improve the haemoglobin levels in pregnancy.

**Conclusions:**

The results indicate that anaemia is alarmingly high among pregnant women in Dhaka city. Maternal anaemia is associated with age, education level, income level, and living area. The results suggest that pregnant women and members of their families should be urgently educated to understand the importance of antenatal care.

## Background

Pregnancy is one of the most and unique periods of women’s life-cycle. It is the most exciting period of expectations and fulfillments; however, it is a condition of great stress because of many anabolic activities that take place during this time and foetal growth takes place with extensive changes in maternal body composition and metabolism [1]. Poor nutritional status during pregnancy is associated with inadequate weight gain, anaemia, retarded foetal growth low birth weight, still births, preterm delivery, intrauterine growth retardation, morbidity and mortality rates [2].

Anaemia is one of the most prevalent nutritional deficiency problems afflicting pregnant women [3], defined by the World Health Organization as haemoglobin levels of <11 g/dL [4]. Anaemia in pregnancy is commonly considered a risk factor for the poor pregnancy outcome and can result in complications that threaten the life of both mother and foetus [5]. It has long been recognized that anaemia is a major public-health problem, especially among poorer segments of the population, in developing countries, such as India, Pakistan, and Bangladesh [6].

The prevalence of anaemia is an important health indicator, and when it is used with other measurements of iron status the haemoglobin concentration can provide information on the severity of iron deficiency [7].

Numerous studies in the developing countries have shown that anaemia, especially iron-deficiency anaemia (IDA), is highly prevalent among pregnant women [8]. Anaemia during pregnancy, particularly iron-deficiency anaemia, continues to be a world-wide concern. It is recognized as the world's most prevalent nutritional disorder, affecting more than two billion people in both developed and developing countries. Pregnant women are particularly at risk of developing IDA with a highest prevalence in South-East Asia [9]. According to the United Nations (UN), 56 % of pregnant women in low-income countries suffer from anaemia compared to 18 % in high-income countries [10]. Its prevalence in pregnancy varies considerably because of the differences in, for example, socioeconomic conditions, lifestyles, and health-seeking behaviours across different cultures. Considering this background, the study was aimed at determining the factors associated with anaemia among a group of pregnant mothers.

## Methods

### Setting

This cross-sectional observational study was conducted at the Marie Stopes Clinic in Dhaka, Bangladesh. The Marie Stopes, a charity organization, is the foremost organization in the SRH (sexual and reproductive health) sector, providing high-quality services, developing and implementing effective programmes especially for the poor and vulnerable people including pregnant women. The service-delivery system of Marie Stopes covers 62 districts, including 43 referral clinics. It has been able to attract patients with different demographic and socioeconomic backgrounds from all over the country. Thus, data generated from this clinic may be considered a fair reflection of general population (pregnant women) of the country.

### Study Population

The study was conducted among 224 pregnant women, aged between 20–35 years with 2^nd^ to 3^rd^ trimester who visited the antenatal clinic of the Marie Stops for routine antenatal check-up. Pregnant women with diabetes mellitus, hypertension, and any congenital haemoglobin disorders were excluded from the study.

### Sample-size

The study revealed that the prevalence of anaemia among pregnant women ranged from 19 % to 50 % [13]. In this study the prevalence was considered 35 % for determining the sample-size using the following formula: n = z^2^q/r^2^p_._ Thus, the sample-size was 204, considering the 10 % non-response and the final sample size for this study was approximately 224.

### Sampling technique

Consecutive sampling technique was applied. Pregnant women were selected who met the inclusion criteria and until either the required sample-size was achieved or the survey period was over during antenatal care period.

### Data-collection

#### Data-collection instrument

A pretested semi-structured questionnaire was used for collecting information on socio-demographic characteristics, reproductive and medical history of the study subjects. Opinion and advice were obtained from teachers, experts from relevant fields, and advisors throughout the initial period of constructing the questionnaire. The questions were constructed in as simple language as possible and a pre-arranged sequence was maintained. The questionnaire was developed in English and then translated into native language Bangla. Pretesting of the questionnaire was performed to gather information on its understandability, time consumed by each question, consistency among related variables and acceptability.

A check list was used for collecting blood samples. The blood test of hemoglobin was performed by the experts in this field. The qualified technician drew venous blood samples from the mothers for the assessment of their anaemia. The reference values of haemoglobin were categorized according to the WHO criteria as: normal (11 g/dL or higher), mild (10–10.9 g/dL), and moderate (7–9.9 g/dL). Mild and moderate levels (<11 g/dL) of haemoglobin were defined as anaemic. [4,12]. Respondents were also asked about history of taking iron supplementation each day.

### Collection of blood sample

2 mg/L EDTA (ethylenediaminetetraacetic acid) vial was collected following all aseptic precautions from the ante-cubital vein using a disposable plastic syringe. Serum was separated by centrifugation (10 minutes) at a rate of 2000 rpm at room temperature; immediately after that blood was allowed to clotting for 30 minutes. Separated serum was allocated in different eppendrof for haemoglobin test. Colorimetric method was used for the measurement of haemoglobin concentration.

### Ethical aspects

Each participant was informed about the research objectives, methods and techniques in detail, and written informed consent was taken from them. Data were collected ensuring the privacy and confidentiality by face-to-face interview. The Ethical Review Committee of the Diabetic Association of Bangladesh (BADAS) and the authority of the antenatal clinic of the Marie Stops of Dhaka, Bangladesh, approved the study.

### Analysis of data

Data were checked thoroughly for consistency and completeness after collection and then cleaned, edited, and verified daily to exclude any error or inconsistency. Statistical analysis was performed using the SPSS software for windows (version 16.0) (SPSS Inc, Chicago, USA). Univariate and bivariate analysis were performed as appropriate. Furthermore, binary logistic regression analysis was done to determine the factors affecting maternal anaemia among the pregnant women. The statistical tests were considered significant at a level of ≤5 % (≤0.05).

## Results

The mean (±SD) age of the study subjects was 26.4 ± 2.81 years, ranging from 20 to 35 years. The majority (64 %) of the women were aged ≥25 years. Most (82 %) women were housewives and 57 % had education from primary to 8^th^ grade. Majority (89 %) was from the urban area and 56 % were from the low-income group (Table 1).

**Table 1:** Sociodemographic and clinical characteristics of the study subjects (n = 224)

Variable	n	%
*Age (*years*)*		
<25	80	36
≥25	144	64
Age [mean(SD)]	26.4 ± 2.81; range 20-35	
*Educational level*		
Illiterate	21	9
Primary to 8^th^ grade	127	57
Secondary and above*	76	34
*Occupation*		
Housewife	183	82
Self-dependent	41	18
*Monthly family income (*Tk**)		
Low income (≤10,000)	126	56
Middle income (10,001-20,000)	62	28
High income (≥20,001)	36	16
*Living area*		
Urban	200	89
Semi-urban	24	11
*Gestational age*		
2 ^nd^ trimester	71	32
3 ^rd^ trimester	153	68
Gravida		
Primi gravida	91	41
Multi gravida	133	59

*above = Higher secondary and graduate; **Tk = (Tk 80 = US$ 1)

**Fig. 1:**
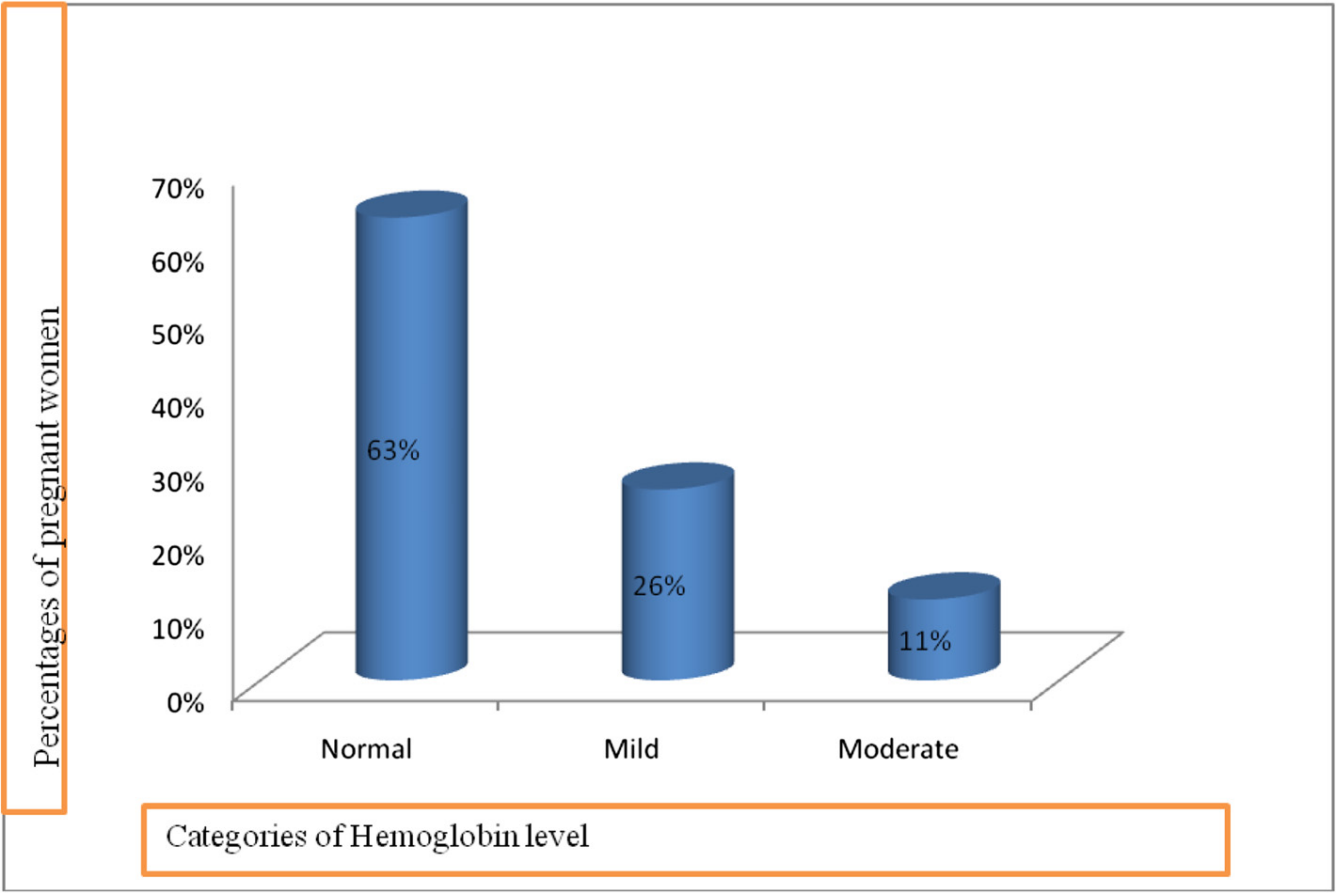
The percentages of pregnant mothers according to their haemoglobin status

About 63 % of the women had normal level of haemoglobin status and 37 % were anaemic (mild 26 % and moderate 11 %) (Fig. 1).

Results of bivariate analyses showed that the socio-demographic characteristics, such as age (p = 0.036), level of education (p = 0.002), income (p = 0.001), and living area (p = 0.031) were significantly associated with maternal anaemia (Table 2).

**Table 2:** Associated determinants among study subjects according to maternal anaemia (n = 224)

Variable	Normal (n = 140) n (%)	Anaemic (n = 59) n (%)	Chi value	p value
Age (years)			4.674	0.031
<25 (n = 51)	44 (55)	36 (45)		
≥25 (n = 128)	100 (69)	44 (31)		
Educational level			8.451	0.015
Illiterate (n = 21)	8 (38)	13 (62)		
Primary to 8^th^ grade (n = 127)	81 (64)	46 (36)		
Secondary and above (n = 76)	55 (72)	21 (28)		
*Occupation*			0.351	0.593
Housewife (n = 183)	116 (63)	67 (37)		
Self-independent (n = 41)	28 (68)	13 (32)		
*Income status* (Tk)			7.993	0.018
Low income (≤10,000) (n = 126)	73 (58)	53 (42)		
Middle income (10,001-20,000) (n = 62)	41 (66)	21 (34)		
High income (≥20,001) (n = 36)	30 (83)	6 (17)		
*Living Area*			5.990	0.014
Urban (n = 200)	134 (67)	66 (33)		
Semi urban (n = 24)	10 (42)	14 (58)		
Trimester			0.499	0.480
Second trimester (n = 71 )	48 (67)	23 (32)		
Third trimester (n = 153)	96 (63)	57 (37)		
Gravida			2.560	0.278
Primi gravida (n = 91)	56 (62)	35 (38)		
Multi gravida (n = 133)	88 (65)	45 (35)		
History of supplementation of iron			0.057	0.811
Yes	131 (65)	72 (35)		
No	13 (62)	8 (38)		

*χ2-test was used. The level of significance at p < 0.005

Further binary logistic regression model was performed to identify the factors affecting maternal anaemia during the pregnancy period. Age (p = 0.006), educational status (primary to 8th grade, p = 0.004; secondary and above, p = 0.002), living area (p = 0.022), and income (p = 0.021) were significantly associated with maternal anaemia. (Table 3)

**Table 3:** Logistic regression analysis considering maternal aneamia as a dependent variable (n = 224)

Independent variable	β	p value	OR	95 % CI for Exp (β)	
				Lower	Upper
Age	-.161	0.006	0.851	0.759	0.955
Education level					
Illiterate	Reference				
Literate	−1.617	0.002	0.199	0.072	0.550
Living area					
Semi-urban^a^	Reference				
Urban	−1.097	0.021	0.334	0.131	0.848
Income	−1.319	0.013	0.268	0.095	0.754
Constant	7.483	0.001	1777.565		

^a^Reference group; β for standardized regression coefficient; Maternal anaemia was taken as a dependent variable whereas others were taken as independent variables. Significant at p value of <0.05; CI = Confidence Interval; OR = Odds ratio

## Discussion

Anaemia is one of the main nutritional deficiency disorders affecting a large proportion of the population, not only in developing but also in industrialized countries. This study found that 37 % of the pregnant women had amaemia. Researchers from various developing countries have reported that 19-50 % of women had the prevalence of anaemia during pregnancy [13]. The prevalence of anaemia in pregnancy has been reported to be 59 % in Bhutan, 65 % in Nepal and 60 % in Sri Lanka [14]. In Bangladesh, the estimated prevalence of anaemia was 50 % [15]. Another study in Bangladesh reported that about 40 % of pregnant women were anaemic [16]. Therefore; it indicates that a large variation in anaemia prevalence exists between countries and possibly within a country. Our result showed the little bit inverse findings compare to the other countries. This may be differing because of regular antenatal check-up and overall improvement of healthcare in the community studied.

Of 224 pregnant women, 140 (63 %) had normal level of haemoglobin, and 84 (37 %) were anaemic in the present study. It can vary considerably due to the differences in socioeconomic conditions, lifestyles and health-seeking behaviours across different parts of the country. Frequency of low haemoglobin levels (42.5 %) was found among pregnant women in the Islamabad and surrounding region of Pakistan [17].

However, the high prevalence of anaemia in Bangladesh and other Asian countries indicates that it is a serious public-health issue that needs attention. A study in Ethiopia showed that the overall prevalence of anaemia was 41.9 %, which is almost similar to our results [18]. An Indian study reported that among 66 pregnant women, 40.92 % had mild, 54.54 % moderate, and 4.54 % severe anaemia [1]. Results of another study showed that, of 51 pregnant anaemic women, 9 (18 %) were mildly, 30 (58 %) moderately, 9 (18 %) severely, and 3 (6 %) were very severely anaemic [19]. Our study has shown converse findings compared to those of Indian studies.

The results of our study showed that level of education of mothers had a significant association with maternal anaemia. This finding is supported by findings of Erlindawati *et al.* in which literacy of women had a significant association with the use of antenatal care services as education has an impact on awareness of use of health services among the population [20].

The findings of this study revealed that the low-income group comprised a higher portion of anaemic patients (n = 53) compared to the high-income group (n = 6), and income was also associated with maternal anaemia. Results of a study in Pakistan showed that patients with a monthly income of less than Rs 5,000 had a haemoglobin value which was 1 g/dL lower than those with a monthly income of greater than Rs 5,000 [21].

In the present study, 37 % of the pregnant women had anaemia in third trimester compared to second trimester. This is probably due to increasing requirement of iron as the pregnancy progresses coupled with the exhaustion of iron stores in most women in the second and third trimesters [22].

The salutary effect of iron supplementation on improvement of haemoglobin levels in pregnancy has been documented in various studies [23], which are more similar to those of our study. Routine prophylaxis of iron is commonly recommended for pregnant women. Initiation of supplementation before conception is needed to reduce maternal anaemia during early pregnancy [24].

Although the sample-size and the study site (clinic) are the two major limitations for the external validation of the findings of our study, it should be mentioned that the organization where the present study was conducted attracts patients with different demographic and socioeconomic backgrounds from all over the country. As the samples were drawn from one clinic, several factors may have impact on the results as to limit its generalizability.

## Conclusions

Based on the findings of the study, it could be concluded that anaemia is alarmingly high among pregnant women and is significantly associated with the socio-demographic factors (age, education, income, and area). Despite this condition, the findings will be useful to our maternal health programme planners and implementers to target and evaluate interventions for the further improvement of health for pregnant mothers of Bangladesh.

## Abbreviations

WHO: World Health Organization
g/dL: Gram/Deciliter
SPSS: Statistical Package for Social Sciences
SD: Standard Deviation
IDA: Iron-Deficiency Anaemia
UN: United Nations
EDTA: Ethylenediaminetetraacetic Acid
BADAS: Diabetic Association of Bangladesh

## References

[1] Sharma P, Nagar R (2013). Hematological profile of anaemic pregnant women attending antenatal hospital. IOSR J Nursing Health Sci.

[2] Kansal B, Guleria K, Agarwal N, Sethi K (2004). Effect of maternal nutritional supplementation on foetal growth parameters and doppler flow velocity in growth restricted foetuses. Ind J Nutr Dietet.

[3] Thangaleela T, Vijayalakshmi P (1994). Impact of anaemia in pregnancy. Ind J Nutr Diet.

[4] Haemoglobin concentrations for the diagnosis of anaemia and assessment of severity. WHO/NMH/NHD/MNM/11.1. VMNIS | Vitamin and Mineral Nutrition Information System. P-VMNIS |. www.who.int/vmnis/indicators/haemoglobin.pdf.

[5] Gregory P, Taslim A, Gergen PJ, Hadden WC, Khan AQ. Health status of the Pakistani population: Health profile and comparison with the United States. Am J Public Health 2001, 91(1):93-8. 10.2105/ajph.91.1.93 PMC1446517 11189831

[6] The prevalence of anaemia in women: a tabulation of available information. 2nd ed. Geneva: World Health Organization, 1992. whqlibdoc.who.int/hq/1992/WHO_MCH_MSM_92.2.pdf.

[7] Assessing the iron status of populations (2007). report of a joint World Health Organization/ Centers for Disease Control and Prevention technical consultation on the assessment of iron status at the population level. 2nd ed.

[8] Toteja GS, Singh P, Dhillon BS, Saxena BN, Ahmed FU, Singh RP (2006). Prevalence of anaemia among pregnant women and adolescent girls in 16 districts of India. Food Nutr Bull.

[9] World Health Organization. Nutrition for health and development. A global agenda for combating malnutrition. Geneva: World Health Organization. 2000. (WHO/NHD/2000.6). whqlibdoc.who.int/hq/2000/WHO_NHD_00.6.pdf

[10] United Nations Administrative Committee on Coordination, Subcommittee on Nutrition (2000). Fourth report on the world nutrition situation: nutrition throughout the life cycle.

[11] FAO, WHO. World Declaration and Plan of Action for Nutrition. International Conference on Nutrition. Rome, Food and Agriculture Organization of the United Nations, December 1992. http://whqlibdoc.who.int/hq/1992/a34303.pdf

[12] WHO, UNICEF, UNU (2001). Iron deficiency anaemia: assessment, prevention and control, a guide for programme managers.

[13] Hyder SMZ, Persson LA, Chowdhury M, Lo¨nnerdal B, Ekstro EC (2004). Anaemia and iron deficiency during pregnancy in rural Bangladesh. Public Health Nutr.

[14] Seshadri S, Gillespie S (1997). Nutritional anaemia in South Asia. Malnutrition in South Asia - A Regional Profile.

[15] HKI (1999). Iron deficiency anemia throughout the lifecycle in rural Bangladesh: National Vitamin A survey 1997–98.

[16] Ahmed F, Mahmuda I, Sattar A, Akhtaruzzaman M (2003). Anaemia and vitamin A deficiency in poor urban pregnant women of Bangladesh. Asia Pacific J Clin Nutr.

[17] Rukhsana A, Nabia T, Malik MA, Mobeen I, Tara J, Shan RR (2009). Low haemoglobin levels, its determinants and associated features among pregnant women in Islamabad and surrounding region. J Pak Med Assoc.

[18] Desalegn S (1993). Prevalence of anaemia in pregnancy in Jima town, southwestern Ethiopia. Ethiop Med J.

[19] Shah AR, Patel ND, Shah MH (2012). Hematological parameters in anemic pregnant women attending the antenatal clinic of rural teaching hospital, *Innovative*. Journal of Medical and Health Science.

[20] Erlindawati, Chompikul J, Isaranurug S (2008). Factors related to the utilization of antenatal care services among pregnant women at health centers in Aceh Besar district, Nanggroe Aceh Darussalam province, Indonesia. J Public Health Dev.

[21] Rukhsana A, Nabia T, Malik MA, Mobeen I, Tara J, Shan RR (2009). Low haemoglobin levels, its determinants and associated features among pregnant women in Islamabad and surrounding region. J Pak Med Assoc.

[22] Barrett JF, Whittaker PG, Williams JG, Lind T (1994). Absorption of nonhaem iron from food during normal pregnancy. Br Med J.

[23] Khambalia AZ, O'Connor DL, Macarthur C, Dupuis A, Zlotkin SH (2009). Periconceptional iron supplementation does not reduce anaemia or improve iron status among pregnant women in rural Bangladesh. Department of Nutritional Sciences, University of Toronto, Toronto, Canada. Am J Clin Nutr.

[24] Quadrat-E-Elahi M, Rahman MM, Momtaz S, Ferdousi MA, Bhuyan FA (2011). Haemoglobin status of pregnant women an analysis of 1804 cases. JAFMC Bangladesh.

